# Mxi1 participates in the progression of lung cancer via the microRNA-300/KLF9/GADD34 Axis

**DOI:** 10.1038/s41419-022-04778-w

**Published:** 2022-05-02

**Authors:** Yujie Lei, Yunchao Huang, Jianbin Lin, Shihui Sun, Keda Che, Junting Shen, Jun Liao, Yangming Chen, Kai Chen, Zhaoxian Lin, Xing Lin

**Affiliations:** 1 Department of Thoracic Surgery, Yunnan Cancer Hospital & The Third Affiliated Hospital of Kunming Medical University & Yunnan Cancer Center, Kunming, 650106 P.R. China; 2 The International Cooperation Key Laboratory of Regional Tumor in High Altitude Area, Yunnan Cancer Hospital & The Third Affiliated Hospital of Kunming Medical University & Yunnan Cancer Center, Kunming, 650106 P.R. China; 3 Department of Thoracic Surgery, Provincial Clinical College of Fujian Medical University & Fujian Provincial Hospital, Fuzhou, 350001 P.R. China

**Keywords:** Cell biology, Cancer

## Abstract

The purpose of the current study was to define the role of MAX interactor 1 (Mxi1) in the pathogenesis of lung cancer and its underlying molecular mechanism. Bioinformatics analysis was performed to identify important regulatory pathway related to lung cancer. Dual luciferase reporter and ChIP assays were adopted to validate the interaction among Mxi1, miR-300 and KLF9. Loss- and gain-of-function studies were conducted to determine the roles of Mxi1, miR-300, and KLF9 in cell proliferation, migration, and invasion in vitro and their effects on myeloid-derived suppressor cell (MDSC) recruitment in vivo. Mxi1 was poorly expressed in lung cancer tissues and cells and its poor expression was associated with poor prognosis. Mxi1 inhibited miR-300 by suppressing its transcription. miR-300 suppressed the expression of KLF9, and KLF9 negatively regulated GADD34 expression in lung cancer cells. Mxi1 or KLF9 elevation or miR-300 repression inhibited lung cancer cell proliferation, as evidenced by reduced Ki67 and PCNA expression, and lowered invasion and migration. In vivo findings revealed that silencing KLF9 induced tumor growth by enhancing MDSC-mediated immunosuppression through upregulation of GADD34. Collectively, these findings suggest that Mxi1 can inhibit lung cancer progression by regulating the miR-300/KLF9 axis and GADD34-mediated immunosuppression.

## Introduction

As the most frequently diagnosed cancer, lung cancer is the leading cause of cancer-related deaths, accounting for more than 1.8 million deaths annually in both sexes worldwide [1]. This disease is usually diagnosed at advanced stages, with local or distant metastasis. At such stages, treatment is less effective, and the mortality rates are higher [2]. Myeloid-derived suppressor cells (MDSCs), immune cells originating from the myeloid lineage, can expand in pathological situations such as cancer and autoimmunity [3–5]. Lung cancer is likely to trigger immunosuppression both locally and systematically, which exacerbates tumor growth and dissemination [6]. Thus, exploring the mechanism of MDSC-mediated immunosuppression in lung cancer development may further our understanding of the pathogenesis of this disease and contribute to improved diagnosis and treatment.

MAX interactor 1 (Mxi1), a member of the mitotic arrest deficient (MAD) family, was first isolated three decades ago, and has subsequently been shown to function as a negative regulator of Myc, which plays a critical role in tumorigenesis [7]. Furthermore, tumor suppressor roles of Mxi1 have been validated in different types of cancers, including prostate cancer, glioblastoma, and lung cancer [8–10]. Importantly, it has also been demonstrated that Mxi1 deficiency exacerbates lung cancer progression [10]. These findings indicate that Mxi1 functions as a tumor suppressor in multiple types of cancers including lung cancer. However, its underlying mechanism is still not elucidated.

In recent years, the critical roles of microRNAs (miRNAs) in the process of tumorigenesis have been highlighted [11]. Accumulating findings have noted the diagnostic and therapeutic potential of miRNAs in oncology [12]. The bioinformatics analysis here revealed that Mxi1 may regulate the expression of miR-300, and a previous research shows that miR-300 exerts a promotive effect on colon cancer progression [13]. Moreover, miR-300 enhances the survival of lung cancer cells in vitro [14]. Furthermore, a recent research has demonstrated that miR-300 upregulation promotes osteosarcoma proliferation and invasion [15]. Thus, it is also possible that miR-300 may also facilitate the tumorigenesis of lung cancer.

In this study, we have endeavored to define the roles of Mxi1 and Mxi1-regulated miR-300 in the initiation/progression of lung cancer using in vitro and in vivo approaches. Moreover, we further identified the downstream molecular activities of these proteins. We proposed a hypothesis that Mxi1 played a suppressive role in MDSC-mediated immunosuppression in lung cancer progression by modulation of miR-300, which consequently regulates the activity of Kruppel-like factor 9 (KLF9)/growth arrest and DNA damage-inducible protein (GADD34) axis. The findings of this study might further our understanding of the pathogenesis of lung cancer and contribute to the improvement of lung cancer diagnosis and treatment.

## Results

### Mxi1 is downregulated in lung cancer tissues and cells and related with poor prognosis

**Fig. 1:**
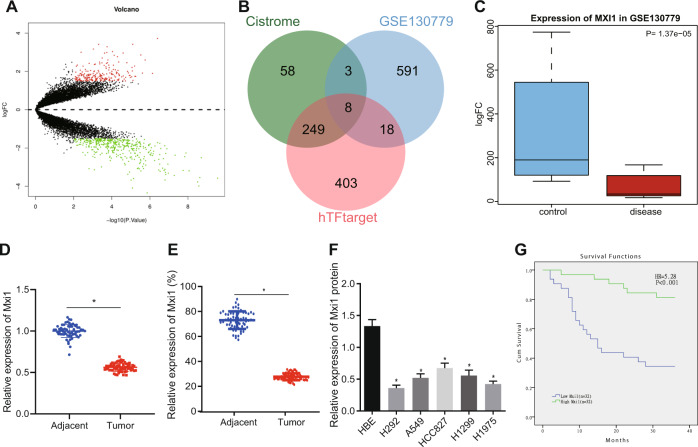
Mxi1 is downregulated in lung cancer tissues and cells and is correlated with poor prognosis. **A** Volcano plots of the gene expression data from GSE130779, red dots: significantly upregulated genes; green dots: significantly downregulated genes; black dots: not significantly changed genes. **B** Venn diagram displaying interactions of DEG of GSE130779 and transcription factors obtained from Cistrome (318) and/or hTFtarget (678). **C** The predicted result of GSE130779 indicating the expression of Mxi1 in lung tumors (red) and normal lung tissues (blue). **D** Result of RT-qPCR detecting the mRNA expression of Mxi1 in lung tumor tissues and adjacent normal tissues (*n* = 64); *p < 0.05, vs. adjacent normal tissues; **E** Representative images of IHC analysis detecting the expression of Mxi1 in lung tumor tissues and adjacent normal tissues (*n* = 64); *p < 0.05, vs. adjacent normal tissues; **F** Western blot analysis evaluating the protein expression of Mxi1 in lung cancer cell liens (H292, A549, HCC827, H1299, and H1975) and normal human bronchial epithelial line HBE; *p < 0.05, vs. HBE cell line. **G** Kaplan–Meier survival analysis comparing the survival difference of patients from Mxi1-high group and Mxi1-low group.

To explore the mechanism of lung cancer pathogenesis, we performed a bioinformatics analysis, which identified 620 differentially expressed genes (DEGs) in the dataset GSE130779 obtained from the Gene Expression Omnibus (GEO) database (Fig. 1A). After intersection of the DEGs with human transcription factors obtained from Cistrome (318) and hTFtarget (678) databases, we obtained eight genes (Fig. 1B). Amongst these, Mxi1 has been considered a potential tumor suppressor with an inhibitory effect on the transcriptional activity of Myc [10]. However, our understanding of Mxi1 in lung cancer remains incomplete. We compared the expression of Mxi1 in lung tumors and normal lungs by analyzing an online-available dataset (GSE130779), which revealed that Mxi1 was poorly expressed in lung tumor (Fig. 1C). We further validated that Mxi1 was downregulated in lung tumors at the mRNA and protein expression (Fig. 1D, E). Moreover, the results of immunohistochemistry analysis revealed that Mxi1 was localized in the nucleus (Fig. 1E). We also determined the expression of Mxi1 in lung cancer cell lines (H292, A549, HCC827, H1299, and H1975) and a normal human bronchial epithelial cell line (HBE). As expected, Mxi1 was poorly expressed in lung cancer cell lines (Figs. 1F and S1A). We then divided lung cancer patients into two groups (low and high-risk groups) based on the expression of Mxi1. According to the results of Kaplan-Meier survival analysis, low Mxi1 expression was significantly related with a dismal overall survival rate among lung cancer patients (Fig. 1G). The above results indicated that Mxi1 was poorly expressed in tumor tissues of lung cancer patients, which indicated poor prognosis in lung cancer patients.

### Mxi1 elevation inhibits lung cancer cell malignant properties

**Fig. 2:**
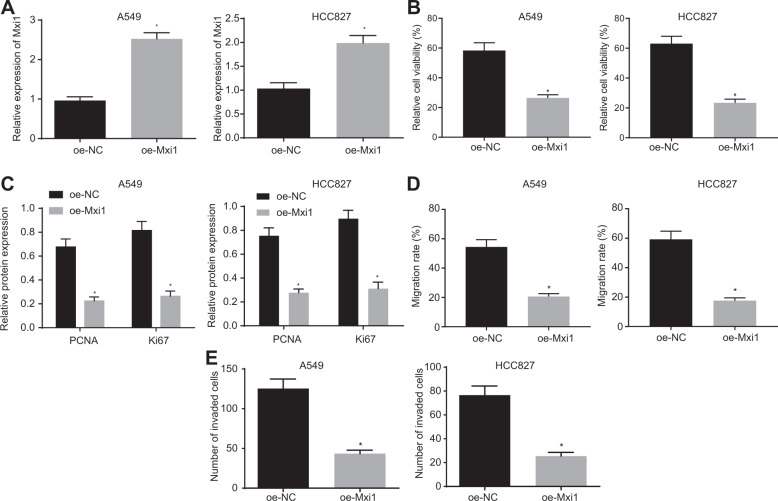
Mxi1 elevation suppresses lung cancer cell proliferation, migration, and invasion. **A** Result of RT-qPCR detecting the mRNA level of Mxi1 in A549 and HCC827 cells. **B** Result of FACS detecting the cell viability of A549 and HCC827 cells with or without Mxi1 overexpression. **C** Representative images and quantitation of Western blot analysis detecting the protein expression of PCNA and Ki67 in A549 and HCC827 with or without Mxi1 overexpression. **D** Result of scratch assay evaluating the migratory potency of A549 cells and HCC827 cells with or without Mxi1 overexpression. **E** Transwell invasion assay detecting the invasion of A549 cells and HCC827 cells with or without Mxi1 overexpression. *p < 0.05, vs. oe-NC. Cell experiments were conducted three times independently.

To define the role of Mxi1 in tumor progression, we altered the expression of Mxi1 in lung cancer cells (A549 and HCC827) and validated the transfection efficiency by RT-qPCR (Fig. 2A). As reflected by fluorescence-activated cell sorting (FACS), upregulated Mxi1 significantly reduced the viability of lung cancer cells (Fig. 2B). Meanwhile, the expression of antigen Ki67 and proliferating cell nuclear antigen (PCNA), which are well-established markers of cell proliferation [16], was measured in the cells using western blot analysis. The result showed that upregulation of Mxi1 brought about downregulated Ki67 and PCNA protein expression (Figs. 2C and S1B). The role of Mxi1 in regulating the mobility of lung cancer cells was also examined by scratch and Transwell assays. The results showed that Mxi1 overexpression resulted in reduced wound closure and lung cancer cell invasion (Figs. 2D, E, and S2A, B). The aforementioned data supported that Mxi1 could suppress the malignant phenotypes of lung cancer cells.

### Mxi1 elevation inhibits the expression of miR-300 at transcription level

**Fig. 3:**
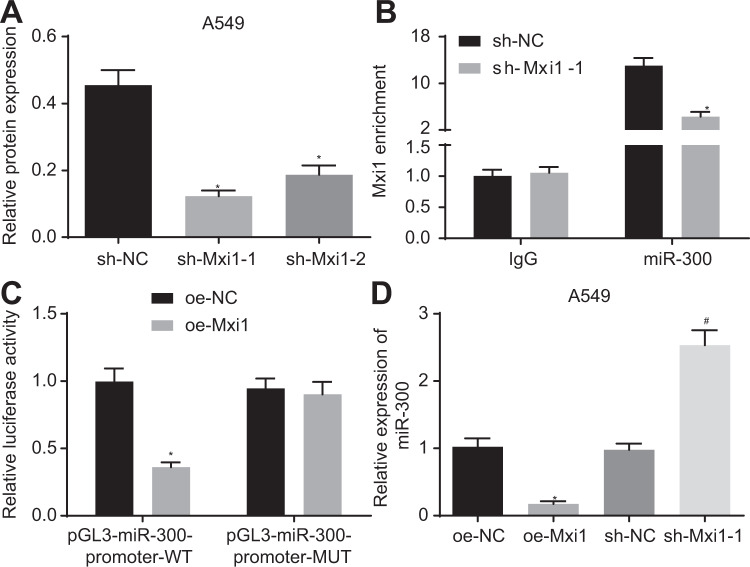
Mxi1 elevation represses miR-300 expression at the transcription level in lung cancer cells. **A** Western blot analysis detecting the protein expression of Mxi1 in A549 cells. *p < 0.05, vs. sh-NC. **B** Result of ChIP assay evaluating the enrichment of Mxi1 at the promoter region of miR-300. *p < 0.05, vs. sh-NC. **C** Result of dual reporter gene assay detecting the interaction between Mxi1 and miR-300 promoter region. *p < 0.05, vs. oe-NC. **D** Result of RT-qPCR detecting the expression of miR-300 under indicated conditions. *p < 0.05, vs. oe-NC; #*p* < 0.05, sh-NC. Cell experiments were conducted three times independently.

It has been reported that Mxi1 suppressed the transcriptional activity of MYC to repress tumor development [10]. Therefore, it is reasonable to postulate that the role of Mxi1 in tumor progression depends, at least in part, in its regulatory effect on gene transcription. According to bioinformatics analysis results, Mxi1 was enriched in the promoter region of miR-300, indicating that it may regulate the expression of miR-300. Meanwhile, recent evidence has suggested that miR-300 promotes the proliferation of lung cancer cells [14]. We altered the expression of Mxi1 *via* RNA interference and the silencing efficiency was validated by western blot analysis. The short hairpin RNA (sh)-(Mxi1 knockdown group 1 (sh-Mxi1-1) with the most significant silencing efficiency of Mxi1 was selected for the subsequent experiments (Figs. 3A and S1C). Moreover, Mxi1 was recruited to the promoter region of miR-300. Meanwhile, our data demonstrated that Mxi1 depletion resulted in the reduced enrichment of Mxi1 at the miR-300 promoter region (Fig. 3B). Furthermore, dual luciferase reporter assay showed that elevated Mxi1 significantly reduced the luciferase activity in cells transfected with reporter plasmid containing miR-300 promoter-wild type (WT). However, Mxi1 overexpression failed to reduce the luciferase activity in cells transfected with reporter plasmids containing miR-300 promoter-mutant type (MUT) (Fig. 3C). To further test the hypothesis that Mxi1 suppresses the expression of miR-300 in lung cancer cells, the transcription level of miR-300 was evaluated by RT-qPCR in A549 cells after the alteration of Mxi1 expression. Results displayed that miR-300 was downregulated by Mxi1 overexpression, but upregulated by Mxi1 silencing (Fig. 3D). The above data indicated that Mxi1 could downregulate the expression of miR-300 in lung cancer cells.

### miR-300 promotes malignant properties of lung cancer cells by inhibiting KLF9

**Fig. 4:**
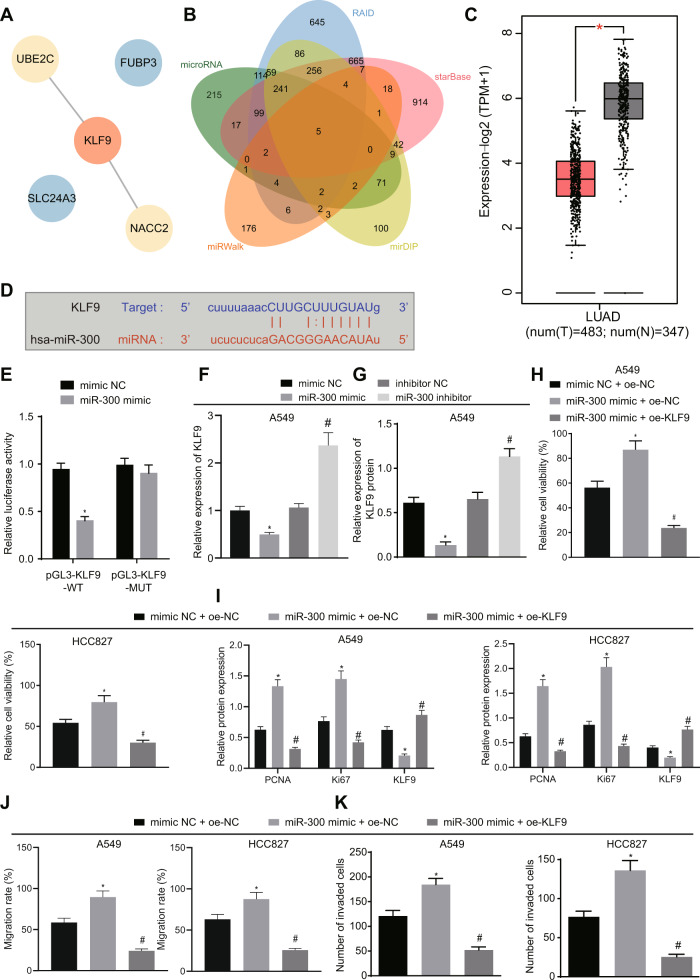
MiR-300 facilitates lung cancer cell proliferation, migration, and invasion by inhibiting KLF9. **A** Downstream genes of miR-300 predicted by microRNA, RAID, starBase, miRDIP, and miRWalk visualized by Venn diagram. Commonly predicted genes are KLF9, NACC2, FUBP3, SLC24A3, and UBE3C. **B** PPI networks established by String database. Red color indicates higher importance while blue color indicates less importance. **C** Relative expression of KLF9 in lung tumors analyzed by GEPIA. Red box: relative expression of KLF9 in lung tumors; gray box: relative expression of KLF9 in normal lung tissues. *p < 0.05. **D** Predicted targeting sequence of miR-300 at the 3′UTR of KLF9 by bioinformatics analysis. **E** Result of dual reporter gene assay evaluating the interaction between miR-300 and 3′UTR of KLF9. *p < 0.05, vs. mimic NC. **F**, **G** Result of RT-qPCR and western blot analysis detecting the expression of KLF9 in cells with miR-300 overexpression or inhibition. *p < 0.05, vs. miR-mimic NC; #*p* < 0.05, vs. inhibitor NC. **H** Result of FACS determining the proliferation of A549 and HCC827 cells under indicated conditions. *p < 0.05, vs. mimic NC + oe-NC; #*p* < 0.05, vs. miR-300 mimic + oe-NC. **I** Western blot analysis evaluating the protein expression of KLF9, Ki67, and PCNA in A549 cells and HCC827 cells under indicated conditions. *p < 0.05, vs. mimic NC + oe-NC; #*p* < 0.05, vs. miR-300 mimic + oe-NC. **J**, **K** Results of scratch assay (**J**) and Transwell invasion assay (**K**) evaluating the migration and invasion of A549 cells and HCC827 cells under indicated conditions. *p < 0.05, vs. mimic NC + oe-NC; #*p* < 0.05, vs. miR-300 mimic + oe-NC. Cell experiments were conducted three times independently.

To better elucidate the underlying mechanism of miR-300 in lung cancer, we initially performed a bioinformatics analysis to detect the potential target genes of miR-300 by searching online databases. Five internet-based miRNA target prediction databases (microRNA, RAID, starBase, miRDIP, and miRWalk) were adopted, and the target genes were predicted accordingly (841, 2197, 2280, 883, and 233 predicted target genes respectively). The potential target genes from each database were compared, yielding the five downstream genes with highest possibility of involvement (Fig. 4A). The protein-protein interaction (PPI) network was analyzed by String and visualized by Cytoscape, which indicated that KLF9 may play a critical role (Fig. 4B). KLF9, a member of KLF family, has been recognized as transcription factor [17]. Members of the KLF family have been demonstrated to possess tumor suppressive and proto-oncogenic effects on tumors [18, 19]. Notably, the inhibitory effects of KLF9 on tumorigenesis have been revealed in multiple types of malignancies [20, 21] including lung tumors [22, 23]. However, the involvement of KLF9 in miR-300-regulated lung cancer progression has not hitherto been investigated. Therefore, we first analyzed the expression of KLF9 in surgical specimens from lung cancer patients *via* the GEPIA website using the dataset obtained from TCGA, which demonstrated that KLF9 was significantly downregulated in lung tumors (Fig. 4C). Therefore, it is possible that miR-300 promotes cancer progression by suppressing KLF9. The potential targeting sequence of miR-300 at the 3′ untranslated region (3′UTR) of KLF9 was predicted by the StarBase database (Fig. 4D) and the interaction between miR-300 and 3′UTR of KLF9 was evaluated by dual reporter gene assay. Results showed that miR-300 overexpression (miR-300 mimic) significantly decreased the luciferase activity in cells transfected with reporter plasmid containing WT 3′UTR of KLF9. However, miR-300 mimic failed to reduce the luciferase activity in cells transfected with KLF9-3′UTR-MUT (Fig. 4E), suggesting that miR-300 could bind to KLF9 3′UTR. This is consistent with a recent study demonstrating that KLF9 is downregulated in lung cancer [24]. Moreover, recent evidence has suggested that KLF9 is associated with cancer cell proliferation [24]. For validation, we altered the expression of miR-300 in A549 cells. We found showed that overexpressing miR-300 resulted in reduced KLF9 expression, while inhibiting miR-300 led to elevated KLF9 in A549 cells (Figs. 4F, G and S1D). These data demonstrated that miR-300 targeted KLF9 and inhibited KLF9 expression in lung cancer cells.

The results of FACS showed that miR-300 overexpression promoted cell proliferation, but this effect was reversed by further overexpressing KLF9 in the cells (Fig. 4H). Meanwhile, miR-300 overexpression resulted in an elevation in Ki67 and PCNA expression but a reduction in KLF9 level, but this effect was abrogated by KLF9 overexpression (Figs. 4I and S1E). As reflected by the scratch assay and Transwell invasion assay, the cells transfected with miR-300 mimic exhibited enhanced mobility, as reflected by promoted wound closure and facilitated cell invasion, whereas these effects were abrogated by KLF9 overexpression (Figs. 4J, K and S2C, D). Altogether, these results suggested that miR-300 could stimulate malignant properties of lung cancer cells by reducing KLF9.

### Mxi1 suppresses lung cancer cells through regulating the miR-300/KLF9 axis

**Fig. 5:**
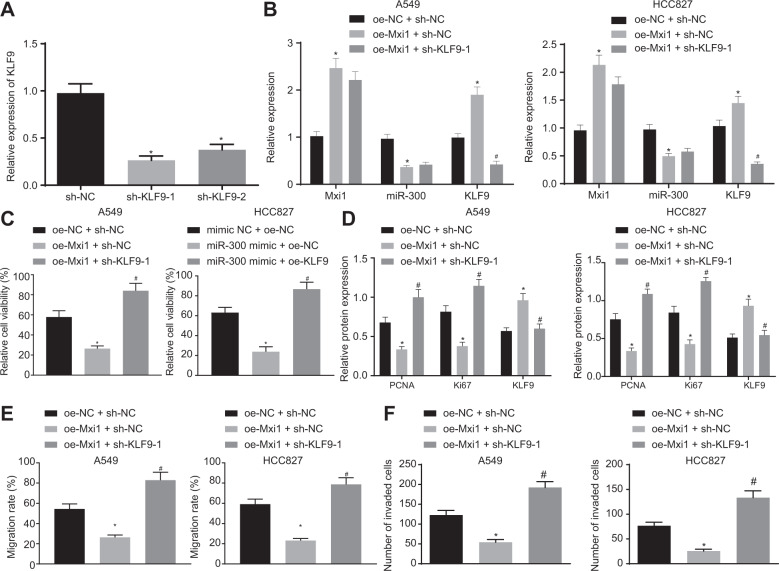
Mxi1 elevation suppresses the malignant phenotype of lung cancer cells through the miR-300/KLF9 axis. **A** Result of RT-qPCR evaluating the knockdown efficiency of KLF9 shRNAs. *p < 0.05, vs. sh-NC. **B** Result of RT-qPCR detecting the expression of Mxi1, miR-300, and KLF9 in A549 and HCC827 cells. **C** Result of FACS evaluating cell viability of A549 cells and HCC827 cells under indicated conditions. **D** Western blot analysis detecting the protein expression of KLF9, Ki67, and PCNA in A549 cells and HCC827 cells under indicated conditions. **E** Scratch assay evaluating the migration ability of A549 cells and HCC827 cells under indicated conditions. **F** Transwell invasion assay investigating the invasion potency of A549 cells and HCC827 cells under indicated conditions. **B**–**F** *p < 0.05, vs. oe-NC + sh-NC, #*p* < 0.05, vs. oe-Mxi1 + sh-NC. Cell experiments were conducted three times independently.

To determine whether miR-300/KLF9 axis was involved in Mxi1-suppressed tumor growth, we modulated the expression of Mxi1, miR-300, and KLF9 separately or in combination. The transfection efficiency was successfully validated by RT-qPCR (Figs. 5A, B and S1F). FACS results showed that Mxi1 overexpression suppressed the cell proliferation, while this suppressive effect of Mxi1 overexpression was impaired by KLF9 silencing (Fig. 5C). As expected, elevated Mxi1 decreased Ki67 and PCNA expression and elevated KLF9 expression, but this effect was reversed by KLF9 depletion (Figs. 5D and S1G). Moreover, results from scratch and Transwell invasion assays demonstrated that Mxi1 overexpression impaired cell mobility as reflected by decreased wound closure (Fig. 5E) and increased invading cells (Fig. 5F), but these effects were counteracted by KLF9 ablation, as reflected by recovered wound closure and cell invasion potency (Figs. 5E, F and S2E, F). Cumulatively, Mxi1 could arrest malignant features of lung cancer cells by regulating the miR-300/KLF9 axis.

### KLF9 elevation suppresses the expression of GADD34 at transcription level

**Fig. 6:**
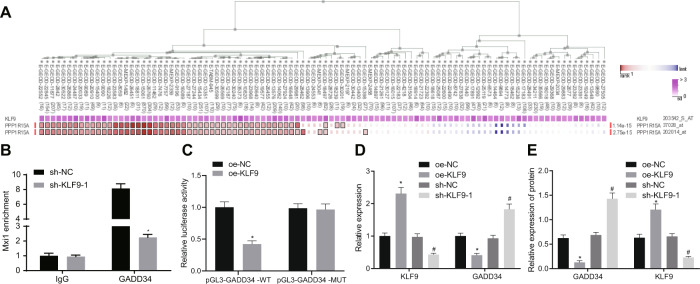
KLF9 suppresses GADD34 expression by inhibiting its transcription. **A** The correlation between KLF9 and GADD34 in lung cancer analyzed by GEPIA database. **B** Result of ChIP assay indicating the enrichment of KLF9 at the promoter region of GADD34. *p < 0.05, vs. sh-NC. **C** Result of dual luciferase reporter assay demonstrating the interaction between KLF9 and the promoter region of GADD34 in HEK293 cells. *p < 0.05, vs. oe-NC; #*p* < 0.05, vs. sh-NC. **D** Result of RT-qPCR evaluating the mRNA expression of GADD34 in lung cancer cells with KLF9 overexpression or silencing. *p < 0.05, vs. oe-NC; #*p* < 0.05, vs. sh-NC. **E** Western blot analysis investigating the protein expression of GADD34 in A549 cells with KLF9 overexpression or depletion. *p < 0.05, vs. oe-NC; #*p* < 0.05, vs. sh-NC. Cell experiments were conducted three times independently.

Previous research has demonstrated that KLF9 interacts with growth arrest and DNA damage-inducible protein (GADD34/PPP1R15A) and suppresses its expression [25]. GADD34 was initially identified in the Chinese hamster ovary cell line [26]. Moreover, other recent evidence has shown that GADD34 serves as a tumor suppressor in liver cancer [27], but promotes lung tumor growth [28]. Therefore, it is possible that KLF9 may suppress tumor progression in a GADD34 dependent manner in lung cancer. To test this speculation, we investigated whether KLF9 targeted GADD34 using an internet-based analysis tool (hTFtarget), which revealed that GADD34 could indeed be targeted by KLF9 in lung cancer (Fig. 6A). Thus, we suppose that KLF9 may regulate the expression of GADD34 by suppressing its transcription. To test this hypothesis, we initially detected the enrichment of KLF9 at the promoter region of GADD34 by ChIP assay. The results showed that KLF9 silencing significantly reduced the recruitment of KLF9 at the promoter region of GADD34 (Fig. 6B). We then conducted dual reporter gene assay to investigate whether KLF9 directly interacted with the promoter region of GADD34 in HEK293 cells. The results showed that overexpression of KLF9 significantly reduced the luciferase activity of GADD34 promoter-WT (Fig. 6C), which indicated that KLF9 could bind to the GADD34 promoter. In the subsequent experiments, we discovered that the transcription of GADD34 was inhibited by KLF9 overexpression but enhanced by KLF9 silencing (Figs. 6D, E and S1H). Taken together, the aforesaid results revealed that KLF9 might reduce the expression of GADD34 at transcription level.

### Knockdown of KLF9 increases GADD34 expression and promotes the tumor growth by inducing the MDSC-mediated immunosuppression

**Fig. 7:**
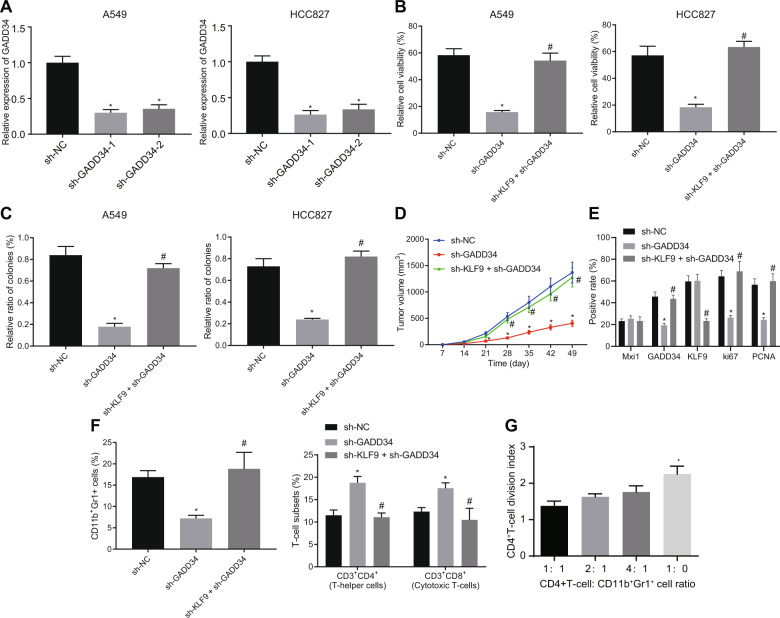
Knockdown of KLF9 results in GADD34 upregulation, which facilitates tumor growth by recruiting MDSCs to induce immunosuppression. **A** Result of RT-qPCR detecting the mRNA expression of GADD34 in A549 and HCC827 cells with GADD34 knockdown. *p < 0.05, sh-GADD34. **B** Result of FACS evaluating proliferation of A549 and HCC827 cells with or without GADD34 knockdown. **C** Result of colony formation assay investigating the proliferation of A549 and HCC827 cells with or without GADD34 knockdown. **D** The size of lung tumor formed by HCC827 cells under indicated conditions. **E** The protein expression of Mxi1, KLF9, GADD34, Ki67 and PCNA protein in the tumor tissues of mice determined with immunohistochemistry. **F** Result of flow cytometry detecting the proportion of the CD11b^+^ Gr^+^ cells, and CD4^+^ cells, as well as CD8^+^ T cells. In panel **B**–**F**, *p < 0.05, vs. sh-NC. #*p* < 0.05, vs. sh-GADD34. **G** Result of CFSE proliferation assay evaluating the cell growth of CD4^+^ cells co-cultured with CD11b^+^ Gr^+^ cells. *n* = 10 for mice following each treatment. Cell experiments were conducted three times independently.

To further investigate the effect of the KLF9/GADD34 axis on the tumor growth of lung cancer, we silenced GADD34 in A549 cells (human origin) and HCC827 cells (human origin) and evaluated the depletion of GADD34 by RT-qPCR. The sh-GADD34-1 with the most significantly silenced GADD34 expression was selected for subsequent analysis (Fig. 7A). In A549 and HCC827 cells, silencing of GADD34 reduced cell viability and proliferation while further silencing of KLF9 augmented cell viability and proliferation (Fig. 7B, C). These data suggested that knockdown of KLF9 could reverse the inhibitory effect of GADD34 knockdown on lung cancer cell viability, thereby promoting lung cancer tumor growth.

Next, we conducted orthotopic implantation of HCC827 cells in C57BL/6 mice to establish a lung tumor model. Tumor growth was significantly impaired in mice receiving GADD34-silenced HCC827 cells while concomitant silencing of KLF9 and GADD34 promoted the tumor growth (Fig. 7D). Moreover, the results of immunohistochemistry demonstrated that the protein expression of GADD34, Ki67 and PCNA was significantly reduced, with no significant difference in that of Mxi1 and KLF9 in tumor tissues of mice receiving GADD34-silenced HCC827 cells relative to that of sh-negative control (NC). Concomitant silencing of KLF9 and GADD34 led to no changes in the Mxi1 protein expression, lower KLF9 protein expression and higher protein expression of GADD34, Ki67 and PCNA than silencing of GADD34 alone (Fig. 7E). The different effects of GADD34 on lung cancer cell growth in vitro and in vivo suggested that GADD34 may promote tumor growth by shaping the tumor microenvironment rather than directly affecting the tumor cell proliferation. To confirm whether this mechanism could also occur under our experimental conditions, we analyzed the component of immune cells in tumor formed by GADD34-silenced cells. The results showed that the number of CD11b^+^ Gr^+^ cells was significantly reduced, while CD4^+^ and CD8^+^ T cells were significantly increased in GADD34-depleted cells. However, the effect of individual silencing of GADD34 was abrogated by simultaneous silencing of KLF9 and GADD34 (Figs. 7F and S3A). CD11b^+^ Gr^+^ cells were then isolated and co-cultured with CFSE-labeled CD4^+^ T cells. As expected, we observed that CD11b^+^ Gr^+^ cells inhibited the proliferation of CD4^+^ T cells as reflected by reduced CFSE fluorescence intensity (Fig. 7G). Taken together, these lines of evidence indicated that knockdown of KLF9 led to an increase in the expression of GADD34, and promoted the accumulation and immunosuppression of MDSCs, thereby promoting the lung cancer growth.

### Mxi1 alleviates the MDSCs-mediated immunosuppression to impair lung cancer progression *via* miR-300/KLF9/GADD34 axis in vivo

To define the role of the Mxi1/miR-300 axis, HCC827 cells that were treated with overexpressed (oe)-NC, oe-Mxi1, oe-Mxi1 + miR-NC, oe-Mxi1 + miR-300 mimic, oe-Mxi1 + sh-NC, or oe-Mxi1 + sh-KLF9 were injected into an orthotropic lung tumor model. We observed that the tumor growth was significantly reduced in mice receiving Mxi1-overexpressing lung cancer cells relative to oe-NC, while opposite effects were observed in mice injected with HCC827 cells expressing oe-Mxi1 + miR-300 mimic or oe-Mxi1 + sh-KLF9 relative to that of oe-Mxi1 + miR-NC or oe-Mxi1 + sh-NC, respectively (Fig. S4A). Moreover, the expression of miR-300 was determined with RT-qPCR, while the expression of Mxi1, GADD34, KLF9, and Ki67 was measured with western blot analysis, the results of which displayed that, compared with oe-NC, overexpression of Mxi1 induced marked reductions in the levels of miR-300, GADD34, Ki67, and PCNA and increased expression of Mxi1 and KLF9. However, opposite effects were observed in mice injected with HCC827 cells expressing oe-Mxi1 + miR-300 mimic relative to that of oe-Mxi1 + miR-NC, while the expression of Mxi1 experienced no marked change. Meanwhile, compared with oe-Mxi1 + sh-NC, increased GADD34, Ki67 and PCNA levels and reduced expression of KLF9 were observed in response to oe-Mxi1 + sh-KLF9, whereas the expression of Mxi1 and miR-300 exhibited no marked change (Fig. S4B, C). As revealed by flow cytometry, the number of CD11b^+^Gr^+^ cells was reduced, while CD4^+^ and CD8^+^ T cells were increased in Mxi1-overexpressed cells, while the opposite effects were observed in mice injected with HCC827 cells expressing oe-Mxi1 + miR-300 mimic or oe-Mxi1 + sh-KLF9 relative to that of oe-Mxi1 + miR-NC or oe-Mxi1 + sh-NC, respectively (Fig. S3B).

## Discussion

**Fig. 8:**
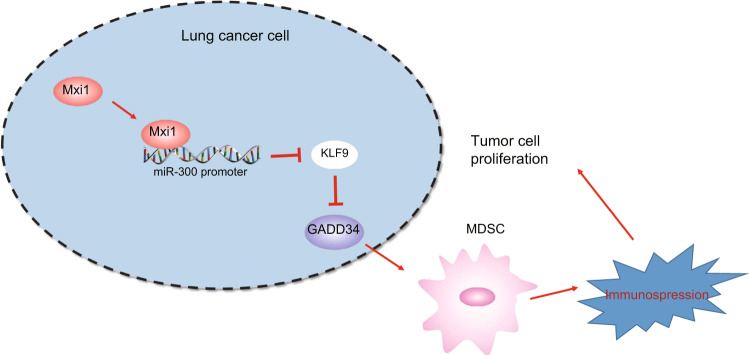
Mechanistic diagram. Mxi1 suppresses miR-300 expression at transcription level, upregulates the expression of KLF9 and reduces GADD34 expression, thus inhibiting immunosuppression of MDSCs and preventing lung tumor progression.

Lung cancer is the leading cause of cancer-related death in the world for all genders [1]. MDSCs are involved in tumor-induced immunosuppression by dramatically inactivating T-cell-triggered antitumor responses, thus contributing to the development of cancer immunotherapies, including that of lung cancer [29]. Thus, the current study was conducted to explore the underlying mechanism of Mxi1 in the MDSC-orchestrated immunosuppression. The results provided evidence indicating that Mxi1 could potentially suppress miR-300 expression at the transcription level to regulate KLF9-mediated GADD34 expression (Fig. 8). This mechanism not only reduced MDSC recruitment in the tumor microenvironment to inhibit immunosuppression in vivo, but also inhibited tumor cell proliferation, invasion, and migration in vitro.

It has been well documented that Mxi1 serves as a negative regulator of Myc and exerts inhibitory action on different malignancies, including lung cancer [8–10]. In this study, we have defined the molecular mechanism by which Mxi1 regulates lung cancer progression, and shown that Mxi1 was significantly downregulated in lung tumors and the downregulation was associated with poor prognosis. This finding is consistent with a recent study [10] focused on identifying the mechanism by which Mxi1 is downregulated in lung cancer. Our study not only confirmed their findings but also furthered our understanding of the role of Mxi1 in the pathogenesis of lung cancer, by demonstrating that Mxi1 elevation inhibited tumor cell proliferation as evidenced by reduced Ki67 and PCNA levels, and lower invasion and migration. These findings are consistent with previous studies [10, 30], which revealed that downregulated Mxi1 facilitated cancer malignancy in lung cancer and brain cancer. Moreover, the correlation of Ki67 and PCNA in cancer cell proliferation has been documented [16, 31], specifically in the context of lung cancer progression [32].

Moreover, in this study, we defined that Mxi1 negatively regulated the expression of miR-300 in lung cancer based on results of our bioinformatics analysis. Consistent with this finding, the role of miR-300 in tumorigenesis has been revealed in different cancers, including lung cancer [13, 14]. It has been well documented that Mxi1 serves as a negative regulator of Myc and exerts an inhibitory effect on lung cancer and other types of malignancy [8–10]. We also found that miR-300 expression was overexpressed in lung tumor tissues, and that miR-300 suppression inhibited tumor cell proliferation as evidenced by reduced Ki67 and PCNA expression, and lower invasion and migration. Consistent with present findings, elevated miR-300 was seen in colorectal cancer tissues, and its elevation promoted the malignant properties of colorectal cancer cells [13].

To further explore how Mxi1-regulated miR-300 was associated with the lung cancer pathogenesis, we analyzed the potential target genes of miR-300, and identified KLF9, which has been reported to be a tumor suppressor [22, 23]. We furthermore revealed that miR-300 targeted KLF9 and decreased the protein expression of KLF9 in lung cancer cells. We also found that KLF9 expression was repressed in lung cancer, and that its repression induced malignant properties in lung cancer cells. An increasing body of evidence has demonstrated that downregulation of KLF9 facilitates lung cancer progression *via* enhancing lung cancer cell malignant properties^,^ [23, 24, 33, 34].

Interestingly, results of the bioinformatics analysis in the current study suggested that KLF9 negatively regulated the transcription of GADD34. Another important finding is that tumors formed from GADD34 deficient cells recruited less MDSCs, as evidenced by reduced numbers of CD11b^+^ Gr^+^ cells and the lesser inhibitory effect of those cells on the proliferation of CD4^+^ T cells. CD11b^+^ Gr^+^ cells are known as a type of MDSCs, which process immunosuppressive activities [35]. Moreover, our findings suggested that the reduced MDSC recruitment inhibited immunosuppression, thus promoting the tumor progression. It has been previously noted that depletion of MDSCs is able to alleviate MDSC-mediated immunosuppression [36]. Interestingly, others have shown that reduced GADD34 levels markedly suppress tumor growth, and decrease the accumulation of MDSCs and T-cells [28].

Our data have demonstrated that Mxi1 overexpression impairs lung cancer cell malignant potentials. Mechanistically, we have revealed that Mxi1 exerts an inhibitory effect on lung cancer progression *via* the miR-300/KLF9/GADD34 axis. Moreover, GADD34 depletion could also inhibit the MDSC-induced immunosuppression, thus inhibiting tumor growth. This study, for the first time, uncovers the underlying mechanism by which Mxi1 suppresses lung cancer progression, imparting an improved understanding of the pathogenesis of lung cancer and providing novel potential therapeutic targets for lung cancer treatment. However, we note certain shortcomings in this study. The number of available patient samples was small, and a larger sample size could mitigate sampling errors. Moreover, Mxi1 may be one among various regulators of miR-300. In addition, the mechanism by which Mxi1 regulates the miR-300/KLF9 axis has yet to be confirmed.

## Materials and methods

### Compliance with ethical standards

This study was conducted with the approval of the ethics committee of Yunnan Cancer Hospital & The Third Affiliated Hospital of Kunming Medical University & Yunnan Cancer Center. All participating patients signed informed consent documents. All human experiments abided by *the Declaration of Helsinki*, and the animal studies were accomplished under a protocol approved by the Institutional Animal Care and Use Committee of Yunnan Cancer Hospital & The Third Affiliated Hospital of Kunming Medical University & Yunnan Cancer Center.

### Patient sample collection

Samples from 64 cases of lung cancer tissues and adjacent normal tissues (at least 5 cm away from the cancer tissues) were collected from lung cancer patients who received surgery in Yunnan Cancer Hospital & The Third Affiliated Hospital of Kunming Medical University & Yunnan Cancer Center from June 2013 to December 2016. Cancer tissues and adjacent normal tissues were fixed with formalin, embedded in paraffin, and cut into sections for further pathological evaluation. All patients had follow-up since the completion of their surgery until December 2019. The clinical records of each participant were updated after each follow-up and overall survival rate was subsequently calculated.

### Bioinformatics analysis

Lung cancer-related microarray dataset GSE130779 (8 normal samples, 8 lung cancer samples) was obtained from GEO database and DEGs were analyzed using R language with the significance threshold at |log2 fold change (FC) | > 1.5, *p* < 0.01. Human transcription factors were obtained from Cistrome and hTFtarget and were intersected with the DEGs obtained from dataset GSE130779, followed by construction of Venn diagram. Potential miRNAs that may be regulated by the transcription factors were predicted by online tool (ChIPBase). The target genes of miRNA were predicted using the following web-based databases: microRNA (conservation > 0.6, mirsvr_score < −0.5), RAID (score > 0.6), starBase (clipExpNum > 3), mirDIP (integrated score > 0.5), and miRWalk (binding > 0.9, energy < −20, and au > 0.45), and then intersected to plot Venn diagram. String (minimum required interaction score: 0.150) was adopted to construct the PPI network of downstream genes. Cytoscape was adopted to investigate the key downstream gene. Targeting sequence of miRNA was predicted by starBase. The expression of target gene and its correlation with miRNA expression were analyzed by GEPIA database. Whether a gene could function as a transcription factor was analyzed by hTFtarget and the co-expression was analyzed with the MEM.

### Immunohistochemistry

Tissues were fixed with 4% paraformaldehyde for 12 h, and dewaxed in xylene followed by serial rehydration (100, 90, 75, ethanol, 3 min each). Sections were boiled in citrate buffer (0.01 M sodium citrate pH 6.0) for 15-20 min. After that, the sections were blocked with goat serum blocking solution (C-0005, Haoran Bio, Shanghai, China) and incubated with primary antibody against Mxi1 (ABP59349, 1: 100, Abbkine, Beijing, China), KLF9 (ab26074, 1: 100; Abcam, Cambridge, UK), GADD34 (ab175355, 1: 100; Abcam), PCNA (ab18197, 1: 100; Abcam), and Ki67 (ab92742, 1: 100; Abcam) overnight at 4 °C. After washing with PBS, the sections were incubated with goat anti-rabbit immunoglobulin G (IgG, ab6785, 1: 1000; Abcam) for 20 min at 37 °C, followed by 20 min of incubation with horseradish peroxidase (HRP)-conjugated streptavidin (0343-10000U, Imunbio) at 37 °C for 20 min. Finally, the sections were incubated with 3, 3′ diaminobenzidine tetrahydrochloride (ST033, Whiga, Guangzhou, China) reagent, stained with hematoxylin (PT001, Bogoo, Shanghai, China) for 1 min, and treated with 1% ammonia to return to blue color. After dehydration in serial ethanol and clearing, the sections were mounted using resin and photographed under a microscope. Five views were randomly selected in each section and 100 cells were counted under each view.

### Cell culture and treatment

Human lung cancer cell lines (H292 [CRL-1848], A549 [CRM-CCL-185], HCC827 [CRL-2868], H1299 [CRL-5803], and H1975 [CRL-5908]) as well as normal human lung bronchial epithelial immortalized cells HBE (CRL-2078) were purchased from American Type Culture Collection (ATCC; Manassas, VA, USA; https://www.atcc.org/). These cells were cultured in Roswell Park Memorial Institute (RPMI)-1640 medium (Gibco, Carlsbad, CA, USA) supplemented with 10% fetal bovine serum (FBS, Gibco) and antibiotics (100 mg/mL streptomycin and 100 U/mL penicillin) in an incubator (Thermo Fisher, Austin, Texas, USA) with 5% CO_2_ at 37 °C.

Lentivirus packaging system was adopted to deliver genes/shRNAs into the cells using LV5-GFP or pSIH1-H1-copGFP vectors as transfer plasmids. oe-NC, oe-Mxi1, oe-KLF9, miR-NC, miR-300, Mxi1 shRNA, KLF9 shRNA, GADD34 shRNA, and sh-NC were synthesized by Gene Pharma (Shanghai, China). Lentivirus was packaged in HEK293T cells (ACS-4500, ATCC), which were cultured in Dulbecco’s modified Eagle’s medium (Gibco) with 10% FBS (Gibco), 10 μg/mL streptomycin and 100 U/ml penicillin for 48 h. Then, the titers were determined.

The cells were divided into 17 groups based on the modulation of the genes in those cells: sh-NC, oe-NC, sh-Mxi1-1, sh-Mxi1-2, mimic NC, miR-300 mimic, miR-300 inhibitor, inhibitor NC, miR-300 mimic + oe-NC, miR-300 mimic + oe-KLF9, oe-Mxi1 + sh-NC, oe-Mxi1 + oe-KLF9, sh-KLF9-1, sh-KLF9-2, sh-GADD34-1, sh-GADD34-2, and sh-KLF9 + sh-GADD34. Both miR-300 mimic and miR-300 inhibitor were purchased and synthesized by Sino Biological Inc. (Beijing, China). Cells were seeded in a six-well plate 24 h before transduction. When reaching about 80% confluence, the cells were transduced according to the instructions of Lipofectamine 2000 reagent (Invitrogen Inc., Carlsbad, CA, USA). After 6 h, the medium was renewed and cells continued to culture for 48 h. The cells were then collected for subsequent experiments. At 48 h after transduction, the expression of related genes in cells was detected by RT-qPCR and Western blot analysis.

### RT-qPCR

Total RNA was isolated from tissue or cells by TRIzol reagent (15596-018, Solarbio, Beijing, China), and RNA concentration was determined. For mRNA detection, total RNA was reversely transcribed into complementary DNA (cDNA) using Reverse Transcription Kit (K1622, Reanta, Beijing, China). For miRNA detection, Poly(A) Tailing Kit (B532451, Shanghai Sangon Biotechnology Co. Ltd., Shanghai, China; containing universal PCR primer R) was used for reverse transcription to obtain the cDNA of miRNA with PolyA tail. RT-qPCR was performed on a ViiA 7 Real-Time PCR system (DAAN GENE, Zhongshan, China) with SYBR/Taq. U6 and glyceraldehyde-3-phosphate dehydrogenase (GAPDH) were used as internal control of miRNA and mRNA, respectively. The primers were synthesized by Takara (Dalian, China) and the sequences are listed in Table S1. The fold change of gene expression was calculated by relative quantification (2^-ΔΔCt^ method).

### Western blot analysis

Cells were lysed with radio-immunoprecipitation assay buffer (Boster, Wuhan, China) containing protease inhibitor and centrifuged. After separation in 10% sodium dodecyl sulfate polyacrylamide gel electrophoresis gels, the membrane was transferred onto polyvinylidene fluoride membranes. The membrane was blocked with 5% bovine serum albumin for 2 h at room temperature followed by incubation with diluted primary antibodies (all from Abcam) overnight at 4 °C: rabbit anti-Mxi1 (ab70594, 1: 500), rabbit anti-KLF9 (ab227920, 1: 500), rabbit anti-GADD34 (ab175355, 1: 500), rabbit anti-PCNA (ab18197, 1: 500), rabbit anti-Ki67 (ab92742, 1: 500), and rabbit anti-GAPDH (ab9485, 1: 500). The next day, the membrane was incubated with HRP-conjugated goat anti-rabbit IgG (ab205719, Abcam, 1: 2,000) and developed with certain reagents. The microscope images were recorded in Vilber Fusion FX5 (Vilber Lourmat, France) and analyzed by Image J1.48 software (National Institutes of Health, Bethesda, USA). The gray value of protein bands was analyzed and used to quantify the relative protein expression.

### Dual luciferase reporter assay

The predicted binding site fragments between the miR-300 promoter and Mix1 and mutant fragments were cloned into the luciferase reporter vector as reporter plasmids miR-300 promoter-WT and miR-300 promoter-MUT. The reporter plasmids were then co-transfected with oe-NC and oe-Mix1 into 293T cells (Oulu Biotecnology, China) to detect whether miR-300 promoter could bind to Mix1. The predicted binding site fragments between GADD34 promoter and KLF9 and mutant fragments were cloned into the luciferase reporter vector as reporter plasmids GADD34 promoter-WT and GADD34 promoter-MUT. The reporter plasmids were co-transfected with oe-NC and oe-KLF9 plasmids into 293T cells (Oulu Biotecnology, China) to determine whether the GADD34 promoter could bind to KLF9. After 24 h, the cells were lysed and centrifuged at 12,000×*g* for 1 min, with the supernatant harvested. The luciferase activity was determined using Dual-Luciferase Reporter Assay System (E1910, Promega, Madison, WI). The relative luciferase activity was calculated as the ratio of relative luciferase activity of Firefly luciferase to that of Renilla luciferase.

### Colony formation assay

Cell proliferation was determined by colony formation assay. In brief, A549 and HCC827 cells and control cells were trypsinized, counted, and seeded into 6-well plates at a density of 1 × 10^3^ cells/well. The cells were cultured for 7-10 days to form colonies. Finally, the colonies were stained by crystal violet and photographed under microscope.

### Detection of T cell proliferation

CD4^+^ CD3^+^ T cells were isolated using mouse T cell isolation kit (R&D system) according to the manufacturer’s instructions. Isolated T cells were stained with 2 μM 5,6-carboxyfluorescein diacetate succinimidyl ester (CFSE) for 10 min and mixed with freshly isolated CD11b^+^ Gr1^+^ MDSC at different ratios (1: 1.2, 1: 4, or 1: 10) and cultured for 3 days. The CFSE fluorescence intensity would reduce by 50% after the cells divided. Finally, the cell division was analyzed by flow cytometry.

### FACS

Cell viability was determined with cell viability detection kit [(Calcein AM, propidium iodide (PI)] (MA0361, Dalian Meilun Biotechnology Co., Ltd., Dalian, China). In brief, cells were harvested, washed with PBS twice, centrifuged and resuspended in 200 μL of binding buffer. The cells were treated with Calcein AM (2 μM) and PI (8 μM), followed by the addition of 300 μL binding buffer. Flow cytometer was finally adopted to detect cell viability at an excitation wavelength of 488 nm.

### Scratch assay

Cell migration was measured by scratch assay as reported previously [37]. In brief, lines were drawn at the bottom of a 6-well plate every 0.5–1 cm using a marker, 5 or more lines were drawn under each well of a six-well plate. Cells at the logarithmic growth phase were seeded into 6-well plates at a density of 5 × 10^5^ cells/well and cultured in medium supplemented with 10% FBS for 24 h. The cells were treated with 1 μg/mL mitomycin C for 1 h, and washed twice with PBS. Then, the scratch was generated with a 10 μL pipette tip and detached cells were removed by washing with PBS. Five randomly selected fields of wound were observed, photographed, and averaged under a microscope at the starting point (0 h) and at 24 h. Cell migration was reflected by wound closure.

### Transwell invasion assay

Cell invasion potency was also investigated by Transwell invasion assay (Transwell chamber containing Matrigel [Becton Dickinson, Franklin Lakes, New Jersey, USA]) as previously described [38]. The number of invaded cells were observed, counted and averaged under an inverted microscopy (CarlZeiss, Jena Germany) in 5 randomly selected fields of each well.

### ChIP assay

Cells were incubated with formaldehyde for 10 min to cross-link DNA and proteins. After that, the DNA-protein complexes were sheared by sonication (UP-250, Scientz, Ningbo, China) into fragments (10 s each time, 10 s intervals, and 15 cycles). After that, cell debris was pelleted by centrifugation (12,000×*g*, 10 min, 4 °C) and the supernatant was divided into tubes. Rabbit anti-Mxi1 (ab70594, Abcam, 1: 100), rabbit anti-KLF9 (ab227920, Abcam, 1: 100), and rabbit anti-IgG (ab109489, Abcam, 1: 300, serving as NC) were added into the supernatants, respectively and incubated overnight at 4 °C. DNA-protein complexes were precipitated by Protein Agarose/Sepharose through centrifugation (12,000×*g*, 5 min). The complexes were then de-crosslinked overnight at 65 °C and DNA fragments were purified by phenol/chloroform. The interaction was investigated by RT-qPCR using specific primers detecting miR-300 and GADD34.

### Mouse model of lung tumor model in mice

Ninety C57BL/6 mice (6-8 weeks old) purchased from the Chinese Academy of Medical Science (Beijing, China) were used for the establishment of an orthotropic model of human lung cancer. All mice were housed in Specific Pathogen Free animal laboratory, with laboratory humidity of 60 to 65% and the temperature of 22-25 °C. All mice had free access to food and water under a 12-hour light and dark cycles. The mice were acclimated for 1 week before the initiation of experiment and their health conditions were monitored. Then, the mice were randomly divided into 9 groups (*n* = 10) based on the cell treatment. A total of 3 × 10^6^ HCC827 cells under different treatment (sh-NC, sh-GADD34-1, sh-KLF9 + sh-GADD34, oe-NC, oe-Mxi1, oe-Mxi1 + miR-NC, oe-Mxi1 + miR-300, oe-Mxi1 + sh-NC, or oe-Mxi1 + sh-KLF9) were injected orthotopically into the middle lobe of right lung of each mouse percutaneously. Six weeks post-injection, the mice were euthanized and tumor size and weight were measured. The tumor tissues were fixed and processed for immunohistochemistry analysis.

### Identification of MDSCs

MDSCs were identified with the instructions provided by the mouse MDSC isolation kit (Cat. No. 130-094-538, Miltenyi Biotec GmbH, Bergisch Gladbach, Germany). In brief, after treatment with FcR Blocking Reagent, cells were stained with biotin-conjugated Gr1 or Ly6G antibody and further labeled with anti-biotin microbeads. The labeled cells were passed through a mass spectrometer magnetic cell separation column. Flow cytometry was adopted to sort out CD11b^+^ Gr^+^ /CD11b^+^ Ly6C^+^ /CD11b^+^ Ly6G^+^ cells to assess the purity of MDSCs (> 90%).

### Statistical analysis

Experimental results were analyzed by SPSS 21.0 software (IBM Corp., Armonk, NY, USA) and data were presented as means ± standard division (SD). Intra-group data were analyzed using paired *t-*test and within-group data were analyzed using unpaired *t-*test. Data among multiple groups were analyzed by one-way analysis of variance (ANOVA) with Turkey’s *post hoc* test. Data among multiple groups at different time points were compared using repeated measures ANOVA with Tukey’s *post hoc* test. Survival rate was analyzed by the Kaplan-Meier method and the difference was compared by Log-rank test. A result was considered as statistically significant when *p* value was <0.05. All experiments were repeated three times.

## Supplementary information

Reproducibility Checklist

## Data Availability

The datasets generated and/or analysed during the current study are available from the corresponding author on reasonable request.
